# Engaging policy in science writing: Patterns and strategies

**DOI:** 10.1371/journal.pone.0220497

**Published:** 2019-08-01

**Authors:** J. B. Ruhl, Stephen M. Posner, Taylor H. Ricketts

**Affiliations:** 1 Vanderbilt University Law School, Nashville, TN, United States of America; 2 COMPASS, Silver Spring, MD, United States of America; 3 Gund Institute for Environment, University of Vermont, Burlington, VT, United States of America; 4 Rubenstein School for Environment and Natural Resources, University of Vermont, Burlington, VT, United States of America; Indiana University Bloomington, UNITED STATES

## Abstract

Many scientific researchers aspire to engage policy in their writing, but translating scientific research and findings into policy discussion often requires an understanding of the institutional complexities of legal and policy processes and actors. To examine how researchers have undertaken that challenge, we developed a set of metrics and applied them to articles published in one of the principal academic publication venues for science and policy—*Science* magazine’s *Policy Forum*. We reviewed each *Policy Forum* article published over a five-year period (2011–15), 220 in all. For each article, we assessed the level of policy content based on presence of a stated policy proposal or position and identification of the relevant policy actors and actions, and recorded attributes such as field of science, field of policy, number of references to legal and policy sources, number of authors from law and policy institutions, and number of citations. We find that a handful of science fields dominate publication frequency, but that all fields have produced publications with high policy engagement. Of the attributes, number of references to law and policy sources is correlated positively with level of engagement, whereas number of law and policy authors was fairly constant across all depths of engagement. Surprisingly, level of policy engagement was negatively correlated with the number of citations an article subsequently received. We offer possible explanations for these results and thoughts for authors, editors, and research institutions interested in facilitating robust engagement of policy in scientific writing.

## Introduction

Bridging the divide between science and policy remains an important and persistent challenge [1, 2] complicated by the many different forms the transmission of scientific findings to policy institutions can take and its many different purposes. While in reality the interface of science and policy emerges out of complex and nuanced social processes [3, 4], there are two basic pathways for science-policy interactions. The first involves a “pull” from policy: policy actors incorporating science into decision making by relying on policy institutions to search for the policy-relevant science they need. For example, a congressional committee could collect and evaluate the relevant available science or commission new scientific studies. The second pathway involves a “push” of information from scientists into the policy arena [5]. For example, scientists could report and connect scientific findings relevant to a given policy issue for the appropriate policy institutions, or produce new policy-relevant science, with the connections to policy made explicit, and transmit it to appropriate policy institutions. In this “push” pathway, a policy institution may not have been seeking the science and thus may need to be convinced it is useful.

There is extensive commentary on the pitfalls inherent in each of these pathways, as policy institutions may cherry-pick for science that supports their decisions and scientists may compromise the integrity of their science when taking policy positions [6, 7]. For our purposes, we assume that scientists engaging in either pathway are using reliable science responsibly and have the communication skills needed to articulate it effectively [8]. From there, we propose that three factors will be highly influential in determining the degree of scientists’ policy engagement evident in their writing: (1) knowledge and understanding of the policy context and relevant institutions; (2) articulation of the policy issue or position; and (3) identification of the policy institutions that can take action on the policy issue.

Here we explore the strategies scientists use in their writing to incorporate these features in their writing if their purpose is to connect research to policy issues and, going further, if they wish to evaluate or recommend policy positions. How far do such scientist writers go to convince their audience that they understand the policy context and the roles of various policy institutions, that they have identified a discrete policy issue to connect or evaluate, and that they have identified which policy institutions can take action? An article that did none of these, but which the author intended to be policy relevant, would passively rely on policy institutions to find the article, determine the policy context, translate the science to policy contexts, and decide what to do about the policy issue and whether they are a viable policy actor. In other words, the article would depend on a “pull” transmission pathway of having the policy institution collect the science. It does not actively engage in a science-to-policy “push” transmission pathway. We do not deem either pathway superior in any resect and recognize that writers may be more comfortable with one over the other. Our goal was to examine the patterns and strategies taken by scientists along this spectrum of writing styles.

To do so, we read and evaluated articles published in one of the principal publication venues for connecting science and policy—*Science* magazine’s *Policy Forum*. The American Association for the Advancement of Science (AAAS), the publisher of *Science*, has informed authors that Policy Forums “present analyses of the policy implications of recent scientific results or studies or discuss the intersection of science and society. Opinion is acceptable but other views should be acknowledged” [9]. Building on this purpose and the status of the venue, we chose to study *Policy Forum* for several reasons. First, *Policy Forum*’s mission statement instruction to authors invites policy engagement—it informs authors that it is a venue where authors can discuss policy implications of science or other intersections of science and policy and it invites authors to express opinion. It does not mandate any particular level of such engagement, however, and thus appears open to authors who wish to engage in either “push” or “pull” styles of writing. We therefore considered *Policy Forum* a convenient source of articles similar in format that would exhibit varying degrees of policy engagement indicia. Second, the editing and, in many cases, outside peer view these articles undergo are likely to result in papers with both strong communication skills and use of reliable science. Third, the impact factor of *Science* is among the highest of all journals, suggesting that scientist writers with a policy engagement goal consistent with the mission statement desire to be published in *Policy Forum*. Lastly, *Policy Forum* provides an established and respected venue where scientists can extend their work into policy domains without fear of being labeled as compromising their scientific standards and integrity.

We stress that these factors pointed us to *Policy Forum* as a useful testing ground for our empirical model, and that we are not singling it out for evaluation. We designed our metrics and variables to apply to any kind of article as a means of assessing the extent of its policy engagement and to measure the relationships with the variables we selected. We seek only to learn more about how authors package their policy-engaged writing.

Also, we do not purport to know how much *Policy Forum* matters to policy institutions and whether it is the most effective venue for scientists who wish to transmit their science to policy institutions. For some fields, such as political science, publishing directly in a journal in that specific discipline may be more effective, and at the very least it would be expected that the author will engage policy. By contrast, *Science* is a peer-reviewed journal covering a broad span of disciplines, and its primary focus is not on policy development. Researchers in many fields, however, do not have access to journals specific to the field that accept policy-oriented writing. Their only option, particularly when they choose to engage in the “push” style of journal writing, is venues like *Policy Forum*. With that premise, we designed an empirical method generalizable to all publication venues to measure the prevalence of “push” and “pull” styles of writing and to identify patterns and strategies authors use along that spectrum.

## Methods

To understand how scientists package their policy-engaged writing, we reviewed each *Policy Forum* article published over a five-year period (2011–15), 220 in all (S1 Table), and developed a coding protocol (S1 Text, S2 Table)). For each article, we measured the overall level of policy engagement based on three criteria: 1) clear articulation of a specific policy position or proposal; 2) clear identification of specific policy actors; and 3) clear identification of specific actions to implement the position or proposal. We classified articles that met all three criteria as “high,” articles that stated a specific position or proposal but did not offer specifics on both actors and actions as “medium,” and articles that did not articulate a policy position or proposal (in which case there necessarily was no actor or action) as “low” (Table 1). Articles classified as high would be more consistent with the “push” style of writing, whereas articles classified as “low” would be more consistent with the “pull” style of writing.

**Table 1:** Method for classifying policy engagement for each article based on three criteria.

*Proposal/position*	*Specific actor*	*Specific action*	Engagement
Yes	Yes	Yes	High
Yes	Yes	No	Medium
Yes	No	Yes	Medium
No	N/A	N/A	Low

For each article, we also classified based on several features and measured several additional factors to indicate how authors demonstrate an understanding of the policy context with which they are engaging. We classified the primary scientific field of an article using the AAAS sections categories [10] and the primary policy field using the University of Toronto’s Atlas of Public Policy and Management policy sectors categories [11] (S1 Text). We counted the number of law or policy experts among authors of each paper. Law or policy experts are defined here as authors affiliated with academic departments such as law and political science, government agencies, law firms, and policy advocacy organizations. We examined the literature cited by each paper and counted the number of references to law and policy publications. Law and policy publications are defined here to include laws, rules, treaties, and other official legal texts, articles in law and policy journals, and books and reports by governments and other organizations that address a policy position or proposal. Finally, we recorded the number of times the article has been cited in the scientific and law literatures (S1 Text, S3 Table).

## Results and discussion

We designed our study to examine the degree to which *Policy Forum* articles overtly display indicia of “push” style policy engagement writing. We do not claim that articles scoring high under our criteria are necessarily more likely to have policy *impact* in the form of governmental or public action. Policy impact is notoriously difficult to predict. Moreover, authors of articles scoring low on our scale may simply not have “push” style of writing as a goal and might engage the policy sector more directly through other means, such as direct engagement with policy makers, boundary organizations [12], or the media [13]. Our scoring measures only the policy engagement of the written work product, not of the authors generally. Overall, however, there is good reason to believe that understanding how articles in venues like *Policy Forum* seek to incorporate policy content can provide insight into how science and society can better connect through science writing. Our results inform that perspective.

### Measuring policy engagement

Slightly less than 27 percent of the articles we reviewed presented a high level of policy engagement indicia, with almost 33 percent ranking medium and just over 40 percent low (S3 Table). For example, Lacetera et al. [14] outlined policies regarding economic rewards to motivate blood donations and suggested specific actions the World Health Organization should take. We ranked this paper as high policy engagement. By contrast, Kennel and Dressler [15] identified key actors in resolving the policy gridlock surrounding NASA’s space science planning, but they called only for a “dialog between government sponsors and the science community.” And Brandt et al. [16] proposed specific actions regarding methane leaks from American natural gas systems, but were not specific about which actors should implement those actions. Because both of these articles leave either proposed actors or actions unclear, we classified them as medium policy engagement. Lastly, Guzman and Stern [17] discuss and demonstrate a method for mapping the founding and growth of entrepreneurial ventures, suggesting it could have “important implications for policy-makers and regional stakeholders,” but they propose no specific policies, actors, or actions. We ranked this article as low policy engagement. We stress again that articles we classified as low policy engagement may nonetheless be of high policy value, but they leave it to policy makers to make those connections.

**Fig 1:**
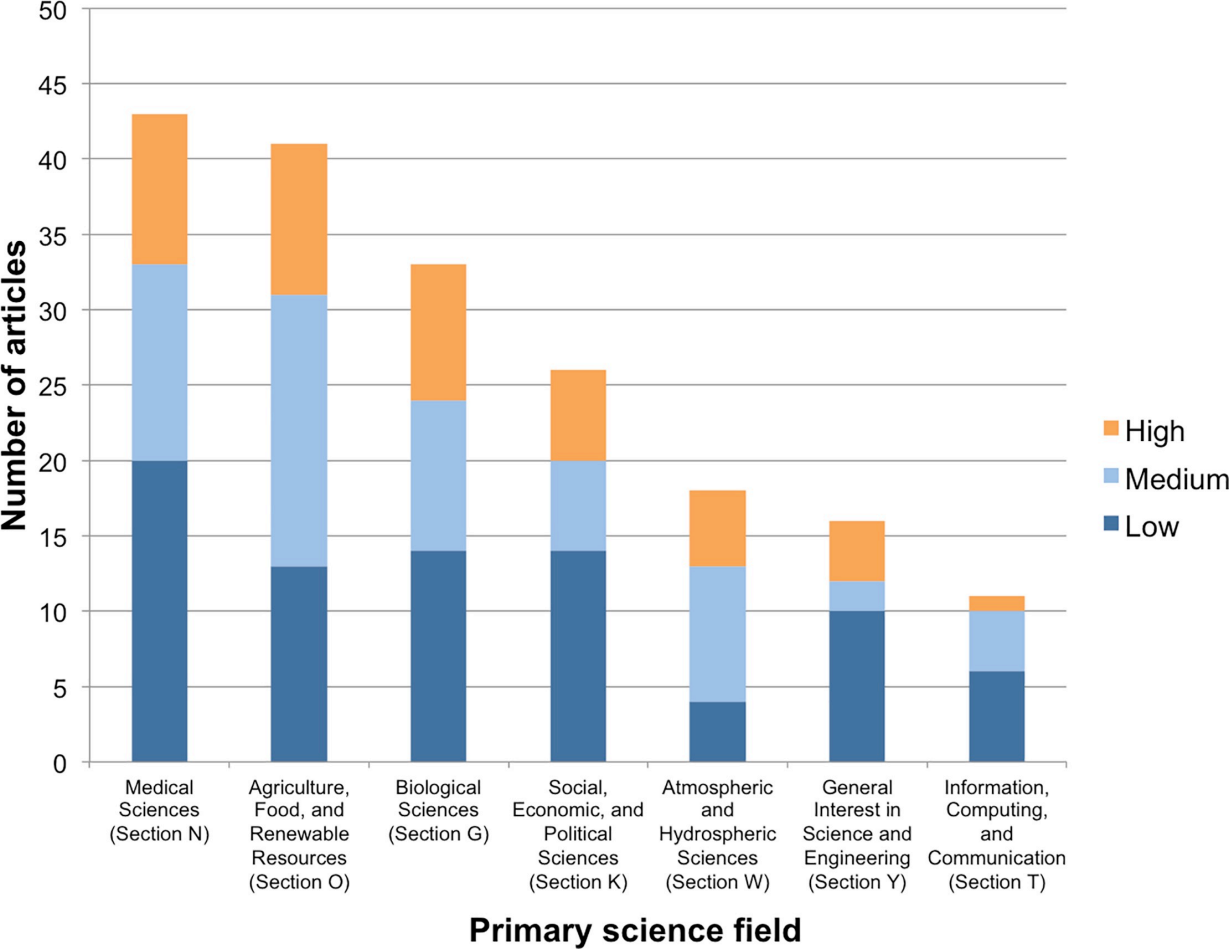
Distribution of articles across primary science fields. Showing only top 7 science fields out of 17.

**Fig 2:**
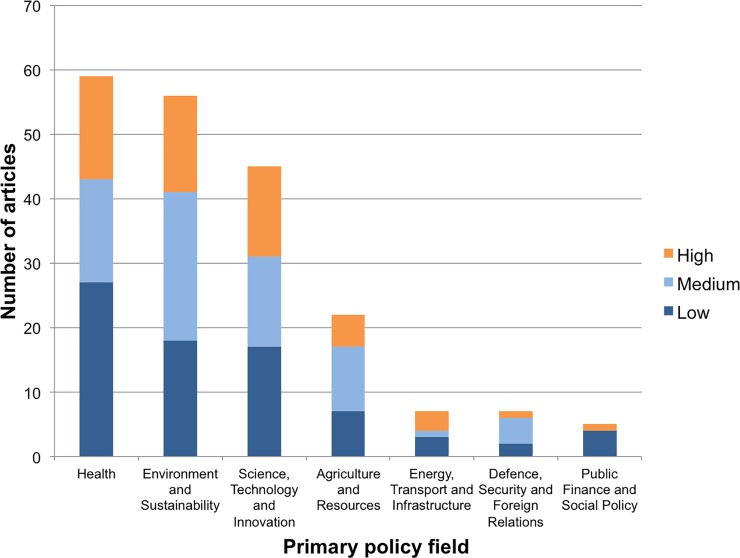
Distribution of articles across primary policy fields. Showing only top 7 policy fields out of 16.

The number of articles is unevenly distributed among scientific and policy fields. The top seven science fields account for 86 percent of all articles (Fig 1) and the top seven policy fields account for 91 percent (Fig 2). Medicine, environment, and agriculture were in the top four fields for both science and policy, accounting for over half of all papers and over half of papers with high policy engagement.

While some fields appear more frequently in *Policy Forum*, fields differ far less in the average policy depth of papers that do appear (Fig 1 and Fig 2). The distribution of articles among high, medium, and low levels of engagement was roughly consistent across science and policy fields. Even severely under-represented fields produced high-engagement articles. There is room in any field of science, in other words, for publishing a policy-engaged article in a high-impact journal.

### Drivers and rewards of policy engagement

**Fig 3:**
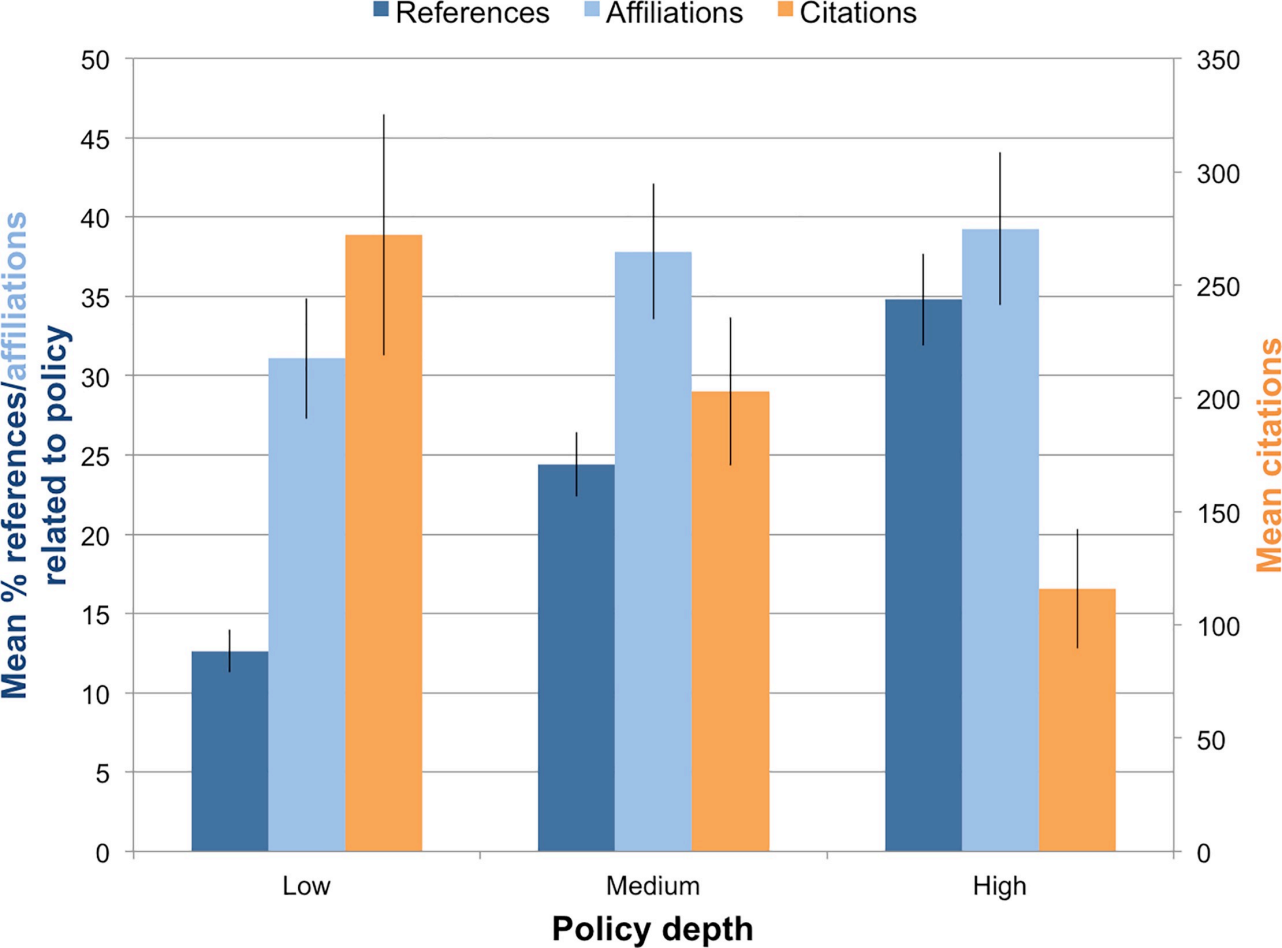
Differences among articles in high, medium, and low levels of policy engagement. Articles in higher categories have a higher percentage of references to policy publications [F(2, 217) = 30.33, p < 0.001], an equivalent percentage of author affiliations related to policy organizations [F(2, 217) = 1.116, p = 0.330], and fewer citations in science journals [F (2, 210) = 3.119, p = 0.046].

The percentage of references to law and policy sources was greater for articles in higher categories of policy engagement (Fig 3). On average, over one third of the references for articles with high policy engagement were to law and policy sources, compared to just under 25 percent for medium articles and 12 percent for low articles. Although referring to more law and policy sources does not necessarily correlate to increase in an article’s level of policy engagement, our finding suggests that high levels of engagement involve knowing, and pointing the reader to, underlying legal and policy references.

We did not find a relationship between levels of policy engagement and policy composition of the research team (Fig 3). The percentage of total policy affiliations was only slightly higher for articles with high policy engagement (39%) than for articles with medium engagement (37%) and low engagement (31%). Focusing only on academic affiliations, however, showed more law and policy affiliations for articles with high policy engagement (23%) than for articles with medium (16%) and low (14%) engagement levels. Overall, the fact that even low engagement articles had over 30 percent of total author affiliations in policy positions suggests that, on average, *Policy Forum* author teams contain a substantial level of policy expertise. Without an expansive study to compare author team compositions of other policy publications, we were unable to determine the degree to which the composition of the author team is an important factor for placement of an article in a venue like *Policy Forum*.

Perhaps ironically, we found that articles with high policy engagement tended to be cited less frequently (Fig 3). Our sample of 220 Policy Forum articles received over 44,000 scientific citations. On average, papers with high policy engagement received over 100 citations, whereas articles with medium engagement received 200 and with low engagement received over 250. This result remained even when we accounted for several positive outliers (e.g., papers with >1000 citations) (S4 Table). This indicates a potential perverse incentive for scientists: articles with higher policy engagement are rewarded with fewer scientific citations. Why? Other scientists, particularly those not interested in engaging policy through their science writing, may find less material in such papers to influence or support their own subsequent research. Another likely explanation is that citation rates are an inadequate measure of a paper’s influence beyond narrow academic publications and audiences. Researchers seeking to engage with policy may simply choose to define impact more broadly, and may disseminate an article they publish in *Policy Forum* directly to policy institutions or other audiences with less concern about citations in journals. Nevertheless, academic citation rates remain an important measure for promotion and success for many researchers.

In contrast to the scientific literature, citations to *Policy Forum* articles in law journals were remarkably low, with an average of under one citation per article and only four articles receiving more than five citations (S3 Table). Citations to law journal articles in *Policy Forum* also are quite low (S3 Table), suggesting a cross-reference disconnect between science and law, the causes of which are beyond the scope of our study but which merit further attention.

## Conclusions: Writing for policy engagement

A skeptic might still ask, why do we need columns like *Policy Forum* at all, and why write for one? The answer is two-fold. First, well-informed policy often must be based on well-supported science. Making those connections—defining the intersections of science and policy—through a venue like *Policy Forum* can provide a valuable source of science for policy makers. Second, when researchers collaborate with policy experts and build their arguments from both scientific and policy literatures, they confront the real-world implications of their work. The resulting articles can influence policy, as well as future research questions themselves, leading to science that is more attuned to the issues, realities, and complexities of the policy world.

Again, we cannot say that publishing in *Policy Forum* or a similar venue is an effective gateway for scientists wishing to engage policy at that level through their journal publications, or that aiming for “high engagement” under our criteria is the best pathway. Our findings suggest, however, that most articles in *Policy Forum* do not exhibit that level of policy engagement (although 60 percent were medium or higher). The reasons why are probably manifold and complicated [18]. No doubt some authors do not wish to engage in “push” style writing, and even some of those who do may believe going too “high” might increase the risk of being perceived as advocating. Others may simply not have the resources or skills needed to achieve high policy engagement in their writing even if that is their goal. It could also be that journal editors and reviewers fall into one or both of these categories. Whatever the reasons, as initiatives in the UK and Australia suggest [19–20], there is no reason to believe they are intractable if the goal is to create an environment in which science researchers inclined to engage in “push” science-to-policy transmission in their writing have access to the resources and training in the skills needed for engaging in as high a level of “honest broker” policy engagement as they wish [8, 21]. So, what do our findings suggest can be done to create that environment? There are potentially important roles for authors, editors, and leaders.

Our study suggests that authors hoping to engage in “push” style writing as their means of transmitting their science across the interface of science and society may wish to consider advancing clear policy positions or proposals and identifying concrete actions for specific actors. The “push” style of writing uses science to support a policy roadmap defining the policy problem, the author’s position or proposal, and who needs to do what about it. Also, our findings suggest that doing this at a high level of policy engagement can be difficult absent meaningful collaboration with law and policy researchers to help identify key references and craft viable policy recommendations.

Editors seeking to promote policy engagement may consider adding columns like *Policy Forum* to their journals. A proliferation of such venues could send a positive message to the relevant scientific communities that policy-engaged writing of all kinds, from “pull” to “push,” is welcome and encouraged. As our results illustrate, that message may not have been communicated (or received) broadly across scientific fields. Editors of such venues could also explore ways more actively to encourage strong contributions from under-represented fields. Broadening beyond the “usual suspects” could help produce more novel and intriguing proposals; examples from our sample include in-depth assessments of the U.S. flood insurance program [22] and of international gene patenting [23].

Leaders of universities and other research institutions can also play a role in providing incentives for and rewarding policy engagement from the scientists they employ. As our analysis illustrates, impact measures too often focus on narrow academic outputs (e.g., papers, citations) instead of policy-relevant outcomes (e.g., changes in awareness, decisions, and policies). Leaders could celebrate policy up-take as much as number of academic publications or citations. And in evaluating promotion and tenure, universities could account more seriously for impact via mentions in media stories, agency reports, congressional testimony, NGO documents, and social media. These additional impact measures could also help journals to evaluate the impact of their science-policy forums. Yet, longstanding attention to citation counts and journal prestige has crowded out such assessments of real-world impact.

The bridge between science and policy will not build itself. There are, of course, many actions needed to build it. Scientists and policy makers can use many different venues for mutual engagement, and much is yet to be learned and trained about which approaches and venues work best [21, 24]. Writing for *Policy Forum* and similar outlets unquestionably can contribute to that overall effort.

## Supporting information

S1 Text Methods. (DOCX)

S1 Table Data. (CSV)

S2 Table Categories of low, medium, and high policy discussion based on our coding methods. (DOCX)

S3 Table Summary of coding results. (DOCX)

S4 Table Results of additional analyses that removed outliers. (DOCX)
