# Supplementary material for: Engaging policy in science writing: Patterns and strategies
Source: PLoS One. 2019 Aug 1;14(8):e0220497. doi: 10.1371/journal.pone.0220497 (PMC6675390; doi:10.1371/journal.pone.0220497)
Supplement: S1 Text — (DOCX) [file pone.0220497.s001.docx]

**S1 Text**

**Methods**

We conducted a systematic review of Policy Forum articles published in Science from 2011-2015 (220 articles total) to determine the extent to which the articles integrated policy affiliations for authors, policy related references, and policy content.

**Policy Forum article search methods**

We used the Vanderbilt Law library to search for Policy Forum articles published in Science during 2011-2015. We initially reviewed 234 articles. We omitted Perspectives articles that were compendiums of multiple short summaries, and articles that did not include a subheading indicating a policy focus. This resulted in 220 total articles that we included in our final coding and analysis (S1 Table).

**Protocol for coding Policy Forum articles**

All three authors coded the same 33 articles. We used this set to compare our coding, develop agreement on coding methods, and synthesize the best coding for these 33 articles. We then divided the remaining articles among the authors for coding (J.B. Ruhl = 60, Stephen M. Posner = 65, Taylor H. Ricketts = 62) and frequently discussed progress to develop consensus on any unclear coding as we proceeded. We coded the following main variables.

*Coder and identifying information for each article*

Article ID is the file name for the pdf file in our database (files are named by date).

*Author information*

We coded each individual affiliation for all authors. One author can fit more than one policy institution category based on multiple affiliations, and we counted each. If multiple authors were affiliated with the same policy institution, we counted each affiliation.

Examples of specific affiliations we encountered and our coding protocol:

- Environmental studies departments are NOT policy departments
- US National Academy of Sciences are a government agency; we reviewed other national science institutes to determine whether they had policy focus
- Medical school only is NOT a policy department; we reviewed medical school centers to determine whether they had policy focus, and if so then we coded as university policy center
- An NGO must be a free-standing organization with no university or other affiliation

*Science and policy sectors*

We coded the AAAS journal category that Science provides above the title of each article. These categories were unique to each article and we did not use them in our analyses.

We coded a primary scientific sector based on the AAAS list of science sections. We also listed other scientific sectors, which we used to discuss articles in question. For similar science categories, we used the following coding protocol:

- Medical Sciences (M) – used if the article had anything to do with human health or the human body, even human genome, cells, etc.
- Agriculture, Food, and Renewable Resources (O) - used this if the article had anything to do with one of the three specified areas
- Biological Sciences (G) – used this as primary only when the focus was exclusively on core biological sciences, ecology, other species, etc.
- Societal Impacts of Science and Engineering (K) – used this if the article argued that practices of science/engineering were having good or bad impacts on people (e.g., data researchers are exposing people to invasions of privacy)
- Social, Economic, and Political Sciences (X) – used this when the article was focused on social sciences and was suggesting ways in which they should change practices, integrate new methods, etc., without suggesting that present practices were having good or bad impacts on people
- General Interest in Science (Y) – used this as last resort if nothing else fit and the article truly was generally about science

We coded a primary policy sector based on the University of Toronto’s Atlas of Public Policy, which is a typology based on the courses taught in over 120 leading Masters of Public Policy and Masters of Public Administration programs. We also listed other policy sectors, which we used to discuss articles in question. For similar policy categories, we used the following coding protocol:

- Agriculture and Resources – used this as primary whenever agriculture/forestry/other resources were the focus
- Environment and Sustainability – used this as primary when there was no Agriculture and Resources focus (e.g., reducing beach erosion or restoring prairie; improving recycling)
- International and Regional Development – used this when there was no specific other policy sector; looked for the specific policy purpose of suggested reforms (e.g., help agriculture; improve health) and coded that as primary

*Policy discussion*

We coded articles as shallow if they did not identify a specific or general actor or policy change. These articles simply identified a policy concern and provided an insubstantial analysis such as “we must do this better.”

We coded articles as deep if they identified a specific actor or a specific policy action (which we defined as a position or proposal). A specific policy position included criticizing or commending a law, statute, or official policy for its societal impacts. A specific policy proposal included identifying best management practices or proposing specific ways any practices should change, even if actors were only generally identified. Of course, categorizing papers required subjective judgment, but the calls were typically clear, and all three authors discussed all borderline cases and then erred in favor of specific ratings. All three authors coded the same initial 33 articles to compare and fine-tune our coding system, with particular attention to developing a common method for coding this variable.

There were only five possible coding outcomes for policy discussion based on the levels of specificity in identifying policy actors and actions (S2 Table). We categorized each possibility into low, medium, or high policy levels.

**S2 Table: Categories of low, medium, and high policy discussion based on our coding methods.**

| **Policy discussion** | **Actors** | **Actions** | **Category** |
| --- | --- | --- | --- |
| None | None | None | Low |
| Shallow | None | None | Low |
| Deep | Specific | General | Medium |
| Deep | General | Specific | Medium |
| Deep | Specific | Specific | High |

*References*

We coded only based on the references contained in the reference and notes list at the end of the article. We did not count any mentions of laws or books in the text of the article.

For total references, we did not count textual notes as a reference if they provided no new references (some listed notes at the end of an article referred to another reference already provided in the list e.g., “8. The government has issued two reports on this issue (2, 7)”).

We coded for three categories of references:

- Specific laws, bills, etc.: These must have been issued by a government entity and be considered legal in effect, not merely a policy analysis, position, or proposal.
- Academic legal or policy journals: These must have been journals (not books, reports, etc.) and must have had a policy focus as at least part of its scope. If uncertain, we reviewed the journal to see how it described itself.
  - We included any reference to a journal with “law review” or “policy analysis” or similar obvious marker terms.
  - We did not assume that “political science” or “social science” or “politics” in title were necessarily policy journals.
- Books, reports, etc.: These must have been published by an NGO, government entity, institution, law firm, university, etc., and it must have been clear from the title or other indicators in the reference that it included policy analysis, position, proposal, or recommendations. We specifically, included a reference if:
  - The title of the book or report used marker words like “policy” “proposal” “reform” “recommendation” “critique” etc.
  - The authoring institution was clearly a policy body (like EPA) or engaged in policy discourse in some instances (e.g., NAS or FAO) AND the report or other document was not clearly simply a data compilation or other non-policy report
    - It was NOT sufficient that the author was a government entity or NGO—it must also have been a policy document as defined above
    - Words in the title such as “outlook” “study” “projection” “handbook” “manual” etc were NOT sufficient— they more likely indicated lack of policy position/proposal

We recorded the reference numbers of the references that corresponded to the three categories, grouped and separated by semi-colon e.g., 1, 4, 7 (referring to specific laws, bills, etc.); none (referring to legal or policy journals); 3, 9 (referring to books, reports, etc.).

**Results**

**S3 Table: Summary of coding results.**

| **Category of Policy Depth** | **Low** | **Medium** | **High** |
| --- | --- | --- | --- |
| **Number of articles**  **(% of total articles)** | 89  (40.5%) | 72  (32.7%) | 59  (26.8%) |
| **Top science sectors** | 1) Medical Sciences  2) (tied) Social, Economic, and Political Sciences  2) (tied) Biological Sciences | 1) Agriculture, Food, and Renewable Resources  2) Medical Sciences  3) Biological Sciences | 1) Medical Sciences  2) Agriculture, Food, and Renewable Resources  3) Biological Sciences |
| **Top policy sectors** | 1) Health  2) Environment and Sustainability  3) Science, Technology, and Innovation | 1) Environment and Sustainability  2) Health  3) Science, Technology, and Innovation | 1) Health  2) Environment and Sustainability  3) Science, Technology, and Innovation |
| **Mean % author affiliations with policy**  **(standard error)** | 31.1  (+/- 3.8) | 37.8  (+/- 4.3) | 39.3  (+/- 4.8) |
| **Mean % references related to policy**  **(standard error)** | 12.6  (+/- 1.3) | 24.4  (+/- 2.0) | 34.8  (+/- 2.9) |
| **Mean # science citations**  **(standard error)** | 272.2  (+/- 53.2) | 203.0  (+/- 32.7) | 115.9  (+/- 26.3) |
| **Mean # law citations**  **(standard error)** | 0.44  (+/- 0.12) | 0.83  (+/- 0.22) | 0.76  (+/- 0.17) |

**Additional analysis of outliers for science citations**

We found that articles in higher categories of policy depth were cited less in science journals. We examined the data and noticed that a small number of articles had many science citations.

To test whether these few outliers were driving our results, we removed outliers in two different separate sub-analyses:

A) we removed 6 articles with greater than 1,000 science citations (a natural break in the spread of the data); and

B) we removed 17 articles with science citations that were greater than 1.5*inter-quartile-range above the 75th percentile (a statistical rule of thumb for defining outliers).

In both cases, we still found a statistically significant result in the same direction as with the full dataset.

**S4 Table: Results of additional analyses on science citations that removed outliers.**

| **Depth** | **Low** | **Medium** | **High** |
| --- | --- | --- | --- |
| **Mean # science citations**  **(standard error)** | 272.2  (+/- 53.2) | 203.0  (+/- 32.7) | 115.9  (+/- 26.3) |
| **Mean # science citations**  ***removed articles > 1000 citations***  **(standard error)** | 180.6  (+/- 24.5) | 174.1  (+/- 26.1) | 115.9  (+/- 26.3) |
| **Mean # science citations**  ***removed articles > 1.5*IQR + 75^th^***  **(standard error)** | 163.5  (+/- 21.2) | 153.4  (+/- 21.7) | 50.26  (+/- 8.7) |
