# Supplementary material for: Engaging policy in science writing: Patterns and strategies
Source: PLoS One. 2019 Aug 1;14(8):e0220497. doi: 10.1371/journal.pone.0220497 (PMC6675390; doi:10.1371/journal.pone.0220497)
Supplement: S2 Table — (DOCX) [file pone.0220497.s003.docx]

**S2 Table: Categories of low, medium, and high policy discussion based on our coding methods.**

| **Policy discussion** | **Actors** | **Actions** | **Category** |
| --- | --- | --- | --- |
| None | None | None | Low |
| Shallow | None | None | Low |
| Deep | Specific | General | Medium |
| Deep | General | Specific | Medium |
| Deep | Specific | Specific | High |
