# Supplementary material for: Engaging policy in science writing: Patterns and strategies
Source: PLoS One. 2019 Aug 1;14(8):e0220497. doi: 10.1371/journal.pone.0220497 (PMC6675390; doi:10.1371/journal.pone.0220497)
Supplement: S3 Table — (DOCX) [file pone.0220497.s004.docx]

**S3 Table: Summary of coding results.**

| **Category of Policy Depth** | **Low** | **Medium** | **High** |
| --- | --- | --- | --- |
| **Number of articles**  **(% of total articles)** | 89  (40.5%) | 72  (32.7%) | 59  (26.8%) |
| **Top science sectors** | 1) Medical Sciences  2) (tied) Social, Economic, and Political Sciences  2) (tied) Biological Sciences | 1) Agriculture, Food, and Renewable Resources  2) Medical Sciences  3) Biological Sciences | 1) Medical Sciences  2) Agriculture, Food, and Renewable Resources  3) Biological Sciences |
| **Top policy sectors** | 1) Health  2) Environment and Sustainability  3) Science, Technology, and Innovation | 1) Environment and Sustainability  2) Health  3) Science, Technology, and Innovation | 1) Health  2) Environment and Sustainability  3) Science, Technology, and Innovation |
| **Mean % author affiliations with policy**  **(standard error)** | 31.1  (+/- 3.8) | 37.8  (+/- 4.3) | 39.3  (+/- 4.8) |
| **Mean % references related to policy**  **(standard error)** | 12.6  (+/- 1.3) | 24.4  (+/- 2.0) | 34.8  (+/- 2.9) |
| **Mean # science citations**  **(standard error)** | 272.2  (+/- 53.2) | 203.0  (+/- 32.7) | 115.9  (+/- 26.3) |
| **Mean # law citations**  **(standard error)** | 0.44  (+/- 0.12) | 0.83  (+/- 0.22) | 0.76  (+/- 0.17) |
