# Supplementary material for: Engaging policy in science writing: Patterns and strategies
Source: PLoS One. 2019 Aug 1;14(8):e0220497. doi: 10.1371/journal.pone.0220497 (PMC6675390; doi:10.1371/journal.pone.0220497)
Supplement: S4 Table — (DOCX) [file pone.0220497.s005.docx]

**S4 Table: Results of additional analyses on science citations that removed outliers.**

| **Depth** | **Low** | **Medium** | **High** |
| --- | --- | --- | --- |
| **Mean # science citations**  **(standard error)** | 272.2  (+/- 53.2) | 203.0  (+/- 32.7) | 115.9  (+/- 26.3) |
| **Mean # science citations**  ***removed articles > 1000 citations***  **(standard error)** | 180.6  (+/- 24.5) | 174.1  (+/- 26.1) | 115.9  (+/- 26.3) |
| **Mean # science citations**  ***removed articles > 1.5*IQR + 75^th^***  **(standard error)** | 163.5  (+/- 21.2) | 153.4  (+/- 21.7) | 50.26  (+/- 8.7) |
